# Traces of an ancient immune system – how an injured arthropod survived 465 million years ago

**DOI:** 10.1038/srep40330

**Published:** 2017-01-11

**Authors:** Brigitte Schoenemann, Euan N. K. Clarkson, Magne Høyberget

**Affiliations:** 1 Department of Neurobiology/Animal Physiology, Biocenter Cologne, Institute of Zoology, University of Cologne, Zülpicherstrasse 47b, D-50674, Köln, Germany; 2 Institute of Biology Education (Zoology), University of Cologne, Herbert-Lewin-Strasse 2, D-50931, Köln, Germany; 3 University of Edinburgh, School of Geosciences, King’s Buildings, West Mains Road, Edinburgh, EH9 3JW, UK; 4 Independent researcher, Mandal, Norway

## Abstract

This report of a severely injured trilobite from the Middle Ordovician (~465 Ma) accords with a number of similar observations of healed lesions observed in trilobites. The uniqueness of the specimen described here is that the character of the repair-mechanisms is reflected by the secondarily built structures, which form the new surface of the ruptured compound eye. Smooth, repaired areas inside the visual surface advert to a clotting principle, rather similar to those of today, and the way in which broken parts of the exoskeleton fused during restoration seem to simulate modern samples. The irregularity and variance of newly inserted visual units indicate the severity of the injury, which, most probably, was caused by a predatory attack, presumably by a cephalopod; these were most likely, the top predators of the Ordovician. Furthermore, the state of the moulted cephalon tells the dramatic struggle of an organism that lived in the Palaeozoic, to survive. In sum the specimen analysed here is evidence of an ancient clotting mechanism not dissimilar to those of today, rapidly preventing any exsanguination and the breakdown of osmoregulation of this marine arthropod.

In the present work we discuss an extremely rare moulted specimen of a trilobite’s cranidium (Fig. 1a), belonging to *Telephina intermedia* (Thorslund)^1^, (Fig. 1b). It comes from dark, limestone concretions (Middle Ordovician Elnes Formation, Heggen Member of Dariwillian age (~465 Ma), in the northern districts of the Oslo Region, and was collected and prepared by M.H.). This moult is extraordinary, because one of the compound eyes clearly shows traces of serious damage which has healed, and the fact that the traces of the healing are represented in a moult means that the injury itself happened before the moulting and that the trilobite had survived the severe injury we describe here. It gives indications about the immune and repair systems of early arthropods, and we discuss possible causes of the injury.

The eyes of *Telephina* have attracted attention in the past and have been described by different authors^2^,^3^,^4^. The holochroal eyes consist of more than 1100 lenses each, arranged in a well developed packing system. In cross section they form an almost semicircular ring, subtending an angle of more or less 110° each in a horizontal sense providing some degree of spatial vision^3^. During their ontogeny^5^ the lenses of *Telephina* develop from a hexagonal to a clearly squared shape, which was first reported by Fortey in 1997, p.403, Fig. 3 6. These appear similar to those of the pelagic trilobite *Pricyclopyge bindosa* (Salter)^7^ and also to those of some living macruran crustaceans, which possess a visual mechanism specialised for relatively dim light conditions. Square facets are typical for reflecting superposition eyes^8^,^9^,^10^,^11^,^12^,^13^,^14^. In such eyes the square lenses form an essential component of an elegant system, in which the four sides of the ommatidia lie at right angles to each other, and act as internally reflecting mirrors. A physiological analysis of this visual system of *Telephina* is a further objective of our present research. It is interesting to note that one of the few previous reports of damage and regeneration in a trilobite eye also concerned *Telephina*^15^. Here, in the present paper, is described an attached left librigena with a well-preserved visual surface. The posterior parts of the visual surface are undamaged, showing the diagonal intersecting rows of lenses typical of *Telephina.* The upper anterior and central parts of the eye, however, have sustained a substantial lesion, an area showing several cavities partially filled with a patch of regenerated lenses. These lenses are circular, and some are somewhat larger than those in the intact regions of the eye. They are regularly arranged in a pattern of some 7 horizontal and 6 near-vertical rows, but this block of regenerated lenses does not accord with the packing system of the undamaged eye but lies at an angle to it.

## Results

**Figure 1:**
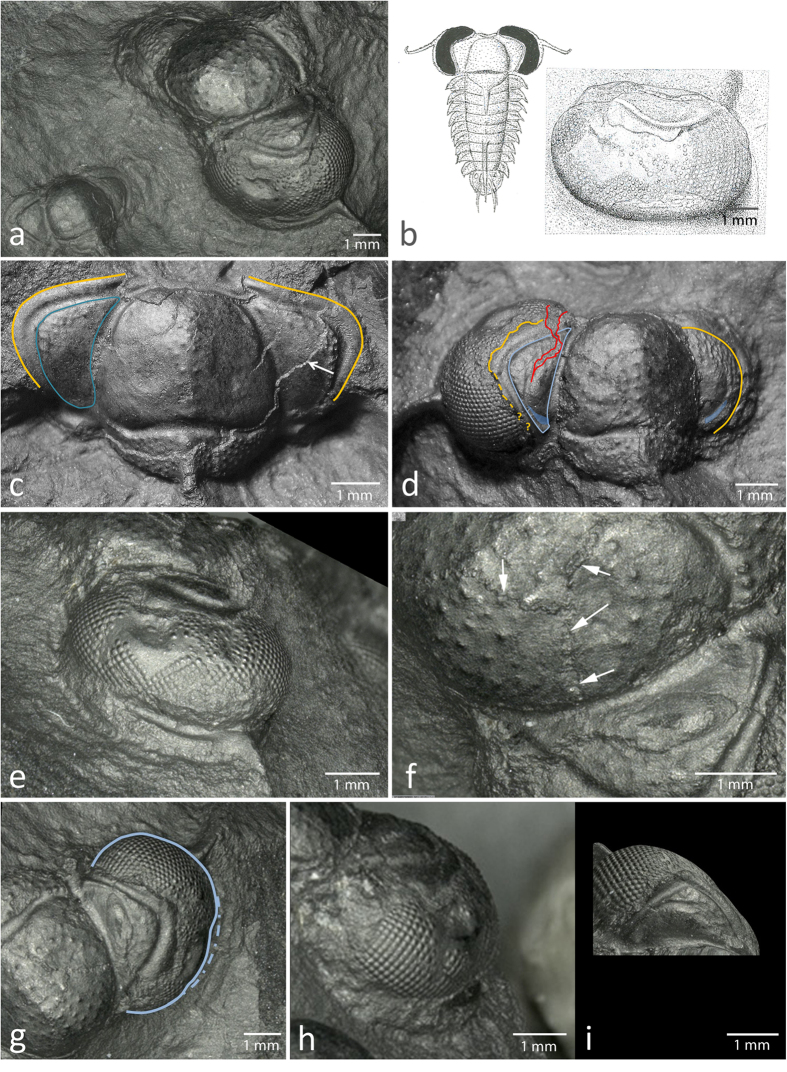
Healed injuries in a cranidium of the trilobite *Telephina intermedia* (Thorslund)^1^. (**a**) Left: moulted cranidium of *Telephina bicuspis* (Angelin)^32^. Right: moulted cranidium of *Telephina intermedia* (Thorslund)^1^. [PMO 231.360, Palaeontological Museum, University of Oslo]; note that the left eye is still attached to the cranidium. (**b**) Reconstruction of *Telephina*, (drawn after Bruton & Høyberget)^3^, and drawing of the compound eye described here. (**c**) Intact moulted cranidium of *T. bicuspis* [PMO 231.361], yellow lines: normal suture between eye and palpebral lobe, blue line: margin of the palpebral area of the fixigena, arrow indicates where the exfoliation of the fixigena begins. To the right of the arrow, the knobbly surface of the exoskeleton is still preserved. (**d**) The same in the moulted cranidium of *T. intermedia.* Yellow lines: sutures between the eye and the palpebral lobe, note its irregular structure on the left side. Blue lines: palebral area. Transparent blue areas: areas of muscle attachments. Red line: Torn moult (comp. text). (**e**) Lateral view of the damaged compound eye of *T. intermedia.* (**f**) Healed cracks in the glabella. Arrows indicate healed and merged seams of the traumatically caused margins of the cracks. (**g**) Deformation of the eye. Blue line: actual shape; dashed line: original shape. (**h**) Same as 1 g, anterior view. (**i**) Same as 1 g, dorsal view.

Figure 1a illustrates the specimen in oblique antero-dorsal view. Normally, as is shown in Fig. 1c, in all species of *Telephina*, the cuticle of the trilobite’s cephalon splits along a facial suture during ecdysis, and separates the free cheeks, including the eyes, from the cranidium. Here, however, it is evident that the left compound eye is still in place (Fig. 1a,d). The exoskeleton is preserved in the right palpebral area of the fixigena, but has been exfoliated in the left palpebral area (Fig. 1d); the left area is therefore an internal mould. The exoskeleton normally shows a knobbly surface with a meshwork of raised lines, while the exfoliated part is smooth (Fig. 1c,d). Such exfoliation is a common occurrence during preparation; the exoskeleton commonly peels off when splitting the rock, leaving a smooth internal surface (Fig. 1c). The knobbly outer structures are typical of every *Telephina* species^3^.

**Figure 2:**
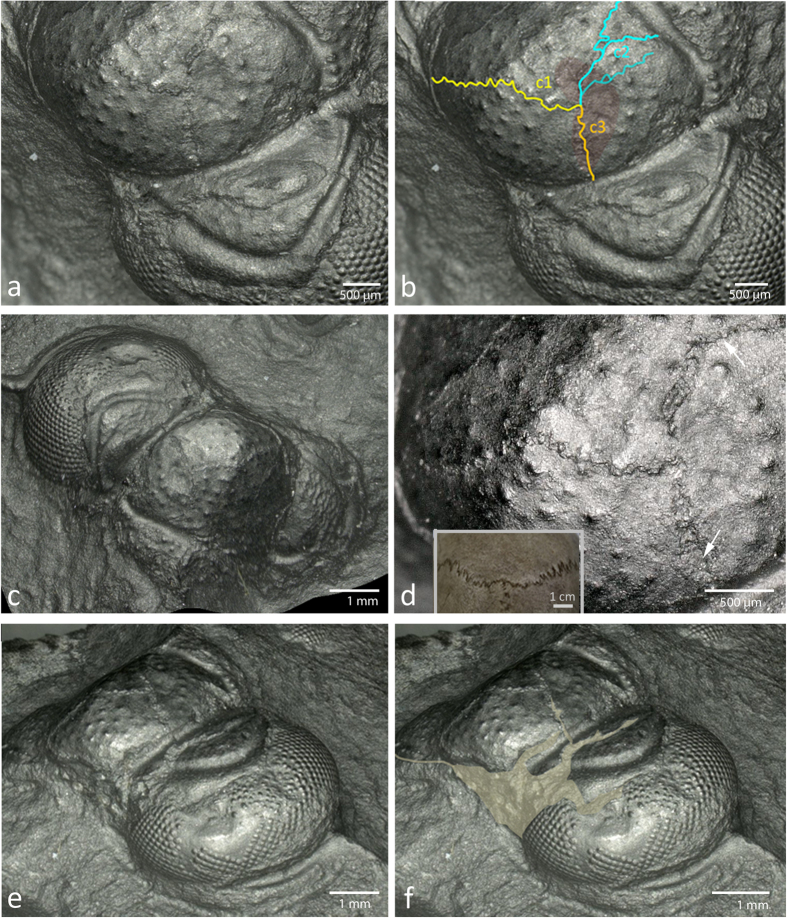
Cracks on the cranidium of *Telephina intermedia* (Thorslund)^1^. (**a**) Cracks on the glabella and in the palpebral area of fixigena. (**b**) The same as (**a**). Transparent red area: traumatic dent on the glabella. c1 (yellow): crack 1 arising from the anterior area of the dent to the right front of the glabella; c2 (blue): cracks from the same origin splitting up backwardly; c3 (orange): crack from the same origin running towards the axial furrow (margin of the palpebral area). (**c**) Cranidium with dents and ruptures seen in dorsal view. (**d**) Aspects of the cracks with healing seams (arrow). Insert: Human skull with *Sutura coronalis* and *Sutura sagittalis*. (**e**) Dents in the visual surface in an anterior-lateral view. (**f**) Secondary rupture (transparent brown area) which established a new fissure to achieve a successful moulting (to leave the old exoskeleton).

**Figure 3:**
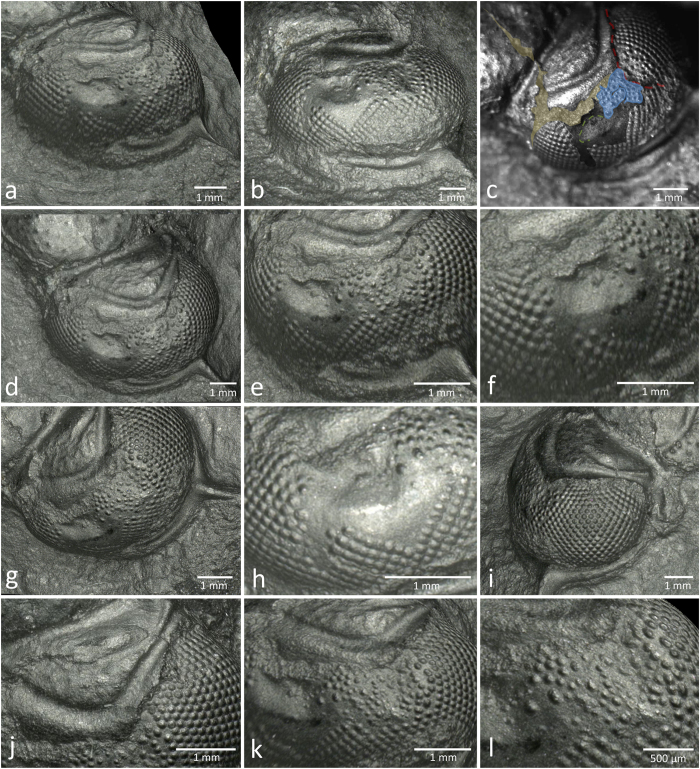
The traumatic dents. (**a**,**b**) Anterior-lateral views of dents and the partly reorganised visual surface. (**c**) Analysis of the visual surface area: transparent brownish field: secondary rupture for successful moulting (comp. 2f). Blue area: reorganised areas of facets. Green line: possible fissure in the torn visual surface. Red line: possible fissure in the torn visual surface. (**d**,**e**) The main two dents and surrounding areas – especially the reestablished field of facets between them. (**f**) Deepest dent inside the visual surface, the triangular field without facets, and the smaller stripe without facets anteriorly. (**g**) Reorganised areas of the visual surface between the two dents. (**h**) Area of repaired compound eye surface without facets. (**i**) View of the posterior part of the compound eye. (**j**) View on top of the broken margin of the palpebral lobe. (**k**) View of the re-established visual surface between the two dents. Note the disorganised pattern of the facets. (**l**) Detail of (**k**).

On the cephalon of the *Telephina* specimen under discussion, three areas with injuries can be discerned. The first is located directly in the center of the head, on the glabella (Figs 1f and 2a–f), the second is a rupture within the fixigena (Fig. 2e,f), and there is a third that lies within the visual surface of the eye itself (Figs 2e,f and 3).

### Injuries

#### Injuries to the dorsal surface of the cephalon

Three long cracks arise from at least two distinct, adjacent dents on middle of the left side of the glabella (transparent red field in Fig. 2b–d). One of the cracks (c1 (yellow) in Fig. 2b) continues anteriorly to the right across the whole glabella and stops at the anterior part of the right palpebral lobe. Another crack (c2 (blue) in Fig. 2b), from the same origin as the latter, extends backwards, splitting after a third of its way and continuing as a Y-shaped fissures towards the occipital furrow, the posterior margin of the cephalon. Furthermore, there is a third fissure from the same origin, extending irregularly towards the back of the glabella, towards the left axial furrow (c3 (orange) in Fig. 2b). There is a thin dark line inside this furrow, resembling a scar, that is to be interpreted as the result of a ‘merging’, or fusion of the margins of the torn glabella (arrows, Fig. 2f). This latter rupture clearly differs from c1 and c2. Both, c1 and c2, show a course of fine wrinkles, while c3 lies at the posterior margin of an irregularly structured surface, which clearly resembles the matrix in which the specimen lies, obviously here the cuticle of the specimen is broken off across this line and widened slightly (Figs 2f and 3d).

The fissures c1-c3 rather probably have to be interpreted as laceration related to the dents in the middle of the glabella, caused by a trauma. Obviously they healed in a dense, interlocking seam and strikingly recall the suture of a vertebrate cranium where the plates of the embryonic skull fuse to a mechanically resistant whole (insert Fig. 2d). Although vertebrates and arthropods are systematically far apart, the principal healing mechanism of fusing broken, flat, hard elements of a skeleton, so important for survival, had already been developed in early trilobites, almost half a billion years ago.

#### Injuries to the fixigena

The suture line between the eye and the palpebral lobe in an undamaged specimen of *T. intermedia* should be distinct and lie in a nearly semi-circular line, comparable to the intact part of the cephalon on the right hand side (Fig. 1d, yellow line). On the left side, however, the border between eye and eye lobe is undulating in the anterior half, and looks crumpled or even curled downwards in the posterior region (Fig. 1d, yellow line, 3a). This suture between the eye lobe and the cranidium appears closed, indicating that this suture has been injured as well, and healed by merging the eye and the cranidium.

This surely had consequences for the moulting process because the exoskeleton, following damage and subsequently suturing, now had been able to open just at the uninjured, and still functional right hand side. Because no carcass, however, lies inside or aside of the exuvia here, the newly grown soft-shelled trilobite obviously succeeded in escaping, probably with vigorous efforts, from the old shell.

Figures 2e,f and 3c show a large and wide distinct crack running across the palpebral lobe and posteriorly throughout the fixigena, where it narrows. The surface of the interior of this crack is identical to the matrix in which the specimen lies, so one may conclude that the cuticle is torn here and that internally the matrix is visible. Along the anterior part of the palpebral lobe, the border has a wavy outline (Figs 2a,b and 3a,d,e). On top of the palpebral lobe we find a kind of lap ripped out, with an unregular outer edge (Figs 2a,d and 3j). Thus, when seen in a lateral view (Figs 2e,f and 3c,e,f,g), there is a rupture arising from the front of the cephalon to the posterior part of the palpebral lobe. A reasonable hypothesis may be that this was torn during the moulting process, caused by the fused palpebral lobe, while the original suture, as described, was merged with the fixigena, and probably was closed in a very stable condition.

It is highly improbable that this rupture is a taphonomic fracture, because the trilobite had no other way of leaving the old cuticle than to tear a secondary ‘suture’.

How far backwards this ripping reached is difficult to see, because the suture line between the palpebral lobe and the eye appears unclear in the posterior part (Figs 1d and 3i,j). The outer rim of the palpebral lobe in the posterior third ends with an irregular line (Fig. 1d, dashed yellow line). Whether it was broken off when the specimen was found or prepared, by cracking open the rock, or whether such irregularity was present already during the life time of the trilobite, cannot be said – in either case it is striking that we find regularly arranged facets where normally a covering rim of a palpebral lobe would be expected. In all trilobites the visual surface grows from top to bottom, and if this standard developmental system was still intact after a violent attack, the missing part may have been compensated for by the lens-generating tissues when brought into contact to the lower parts of the fixigena.

Another, perhaps even more plausible interpretation is, that the suture was functioning very well in the undamaged posterior part, resulting in a natural splitting and overlapping of the palpebral lobe and the eye during the struggle of moulting. The width of the crack in the front corresponds to the size of this lap posteriorly. The posterior part of the palpebral lobe thus probably became broken off when splitting the rock, uncovering the hidden top of the eye. Despite the rupture in the anterior part and the natural splitting along the undamaged suture in the posterior part, anteriorly the eye and librigena were still attached to the glabella.

Another point arises when the smooth muscle scar on the posterior part of the fixigena is considered (blue falcate field in Fig. 1d). This is very distinct in all *Telephina* species. On the right, intact fixigena, as part of the moulted cephalon, the scar is distinctly bent, linear-shaped small furrow. When compared to this, the equivalent scar on the left fixigena appears more or less crescently shaped and slightly shortened. That this scar was altered as a result of the injuries, indicates a complex healing process underneath. Obviously, the attachment of the underlying muscle was changed by this, and the ability to move the ventral appendages in the mouth region, which were probably used for food gathering, the trilobite must have been at least slightly affected.

#### Injuries to the compound eye

The third type of lesion seems to be the most severe of the injuries, which had the effect of changing the entire shape of the compound eye, causing it to lose its semicircular outline (Fig. 1g).

We find, three-dimensionally, at least two areas of dents inside the visual surface (Fig. 3a–i), disturbed patterns of the facet lattice (Figs 1a,b,e,h, 2e,f and 3), and even areas without facets (Fig. 3a,b,e,f,h). These are areas, completely smooth, which more or less follow the shape of the former compound eye´s surface, indicate an active healing process in the background, restoring the original functional shape.

Figure 3b,g,h show a significant, horizontally oval dent in the central anterior part of the visual surface below the rupture discussed before, and another (Fig. 3b,e,g,i) on the upper, more posterior part of the compound eye. The former, deep injury, is quite extensive and may have caused a stub/tear off of the visual surface, which lead to the anomalies of the outline of the palpebral lobe with the consequences discussed before, such as the closed suture line between the eye and palpebral lobe following the rupture.

As mentioned, posterior to this a second, slightly smaller dent can be observed (Fig. 3b,e,g,i). Figure 3c shows that here also a fissure may have occurred, which obviously healed (red line Fig. 3c). The space along the formerly tattered margin between the two dents was filled by irregularly arranged facets of different sizes and shapes, many of them are much larger than the originals. This is somewhat surprising, because larger lenses capture more light than smaller ones, and thus support more sensitive visual units than in the rest of the intact visual surface. It might be, however, that some unknown mechanism made these larger and irregularly positioned units to compensate for ‘lost’ light, which otherwise would have been captured by the now perished lenses. Another option is that the replaced sensory system simply did not work as effectively as the previous one, and thus needed more light. The same may have been true for the large regenerated lenses in the specimen described by Isberg (1917, p. 594–595)^15^.

The space between the two injuries shows also a highly anomalous ordering of the lenses (Fig. 3a–e,g,k,l). This ordering is very irregular, and the size of the lenses varies, some are larger than the original ones, others (especially in the central part) are smaller. The distances between the lenses show nothing of the normal lattice, though a rough pattern can be made out (Fig. 3c in blue). These findings surely show, that the moment of the injury took place before the moulting process, because to generate these new patterns, and to build new visual units with lenses takes time. Secondly, it is evident that the injuries are not post mortal, as for example the result of scavenging, because no traces of regeneration and reorganisation would to be observed in that case. Thus, it was an ‘old’ injury formed during the active life of the trilobite, which probably happened quite independently of any moulting process.

The most important area for understanding what had happened is the part of the visual surface where there is no trace of any patterned structure (Figs 1d, 2e,f and 3b,c,e,f,h). This region lies below the more extended, anterior dent inside the surface of the compound eye. Only indications of 5–6 small regenerated lenses are discernable at the posterior rim of the dent, the rest of the surface is very smooth. The shape of these lenses is rounded, not hexagonal or square as might be expected. There is another smooth and slender stripe in front of this area (Fig. 3b,f,h). Remarkably, in its upper part, 3–4 small lenses were reestablished and link both sides of this smooth stripe (Fig. 3b,h). The shape of these elements, however, is not as well expressed compared with other regenerated lenses.

The whole aspect of the smooth area below the extended dent resembles an opened zip, where the lenses are torn to the left and right, while the interspace is smooth. The most important part is the area between these segregated lines of lenses. It is striking that here no further facets were regenerated, the surface is absolutely smooth (Fig. 3h). This area seems to be the most instructive of the whole system of injuries. Here it is evident that the injured visual surface has been healed and reconstructed (more or less) in shape, but without any regeneration of lenses. It is probably the case that all of the deeper-lying centres, capable of generating visual units, had been destroyed. Instead, the immune system produced a shape-conserving substitute, guaranteeing a functional overall shape of the compound eye and closing off and protecting the whole area so that as far as possible, a normal metabolism could continue.

#### Healing process

Much is known about the healing processes in living panarthropods. Following an injury, generally an increase of plasmocysts in the haemolymph is observed, sometimes even at a factor of 10. After a lesion of the hypodermis granulocysts secrete a substance, which together with different humoral factors form a kind of gel, which together with haemocysts embraces the wound and finally builds a wound-plug, in other words clotting. An important part of this system is the enzyme phenyloxidase. It operates together with particular coagulation proteins, which differ in the various groups of arthropods, to clot certain proteins of the haemolymph, and forming a network not dissimilar to the generation of fibrin in vertebrates. This extracellular gel, produced as a result of the clotting-process, finally is covered by a thin epithelium^16^,^17^,^18^, and well may offer an interpretation of the smooth surfaces of the healed part below the large anterior extended dent and the small strip in front of it.

What we see in this specimen of *Telephina intermedia* (Thorslund)^1^, therefore can be interpreted as the result of an ancient, and successful clotting process. We do not know in detail what the contributing proteins were, but our observations suggest a principle analogous to that of today. There are several examples of this: the closed cracks on the glabella, the repaired injuries of the posterior dent, the mixture of tissue relicts fused to an irregular area, and one area equipped with regenerated visual units, the closed indented surfaces of the extended injury, the ‘attempt’ to reconstruct at least parts of the visual surface with new lenses, and the smooth now substituted former visual surface, with no traces of lenses at all. They all are fused to a ‘repaired’ compound eye, which could be moulted, and together with the successfully healed cracks in the glabella, in close similarity to modern clotting processes, they probably witness that similar processes operated in an Ordovician arthropod, some 465 million years ago.

## Discussion

What is seen here is surely the result of an injury, and it is so extensive that in our estimation it is very likely to be the result of a predatorial attack (even cannibalism). Although an alternative explanation might be an accidental blow by falling debris for example. The injuries sustained, however, are much more consistent with a predatory attack – the exoskeleton (calcium carbonate in organic base) would have had at least some elasticity and therefore it would have rebounded rather than breaking. Other possibilities to cause this injury might include genetic causes, but the injuries are unilateral and asymmetric, parasitism – but the injuries are markedly irregular, or disease, which probably would not result in any cracking and subsequent regeneration as found here. So, trauma by a predator is almost certainly the cause for these lesions. It is very interesting to see how some of the lenses have been regenerated after this injury. The regenerated lenses are rounded in form, with a tendency to be scattered irregularly, retaining the original order just where indicated. They are independent in size, which may be a consequence of disruption of the ommatidial context. Clearly the attack was traumatic for the trilobite, and a strong challenge for the osmotic management and internal pressure conditions. Additionally, because of the risk of dying by exsanguination the injury must have been closed very quickly. After the successful healing, in this case another strong challenge might have been the fused suture, because the pre-ecdysial, soft-shelled trilobite will have developed underneath its exoskeleton but may not have been able to escape from the old carapace without tearing it with strong force.

**Figure 4:**
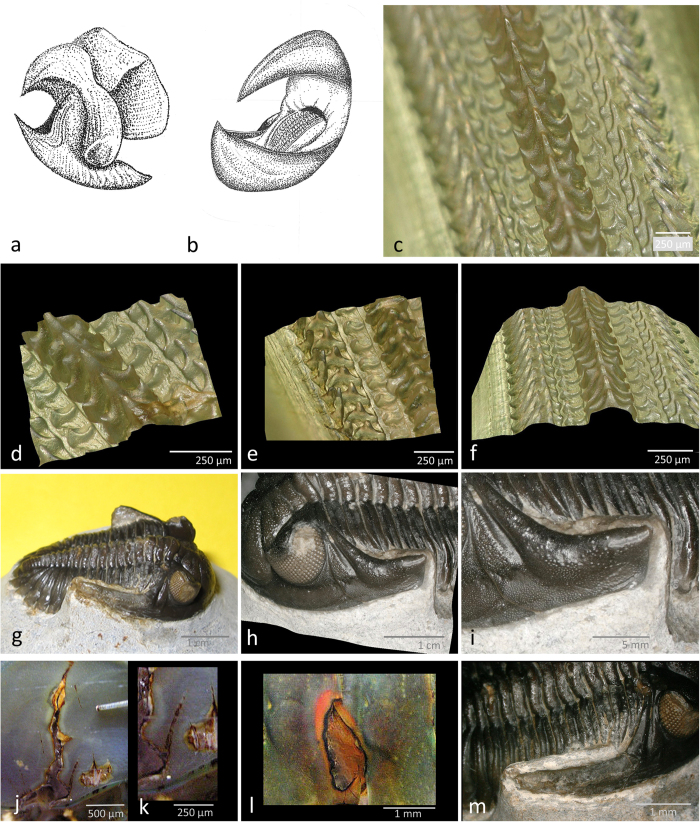
Drawing of the parrot-like beak of a cephalopod mouth. (**a**,**b**) Beak-like mouthpart, (**b**) with radula inside. (**c**) Surface of the radula. (**d**) Central elements of the radula. (**e**) Lateral rasp-like hooks of the radula. (**f**) 3D-relief of the radula. Regenerated genal spine of *Hollardops mericristata* (Le Maître)^31^, [PMO 231.467]. (**g**) *Hollardops mericristata* (Le Maître,)^31^. (**h**)The injured and shorter, regenerated left spine. (**i**) Detail of (**h**,**j**) Injured, broken carapace of a shrimp. (**k**) Detail of (**j**), note the fused seam inside of the healed crack. (**l**) Healing planar area of a lesion in the carapace of a shrimp. Note the thin epithelian tissue covering the wound. [(**j**-**l**) courtesy Wolfinger see also^34^,^35^].

The most likely candidates for effecting these lesions are cephalopods. The supposed pelagic mode of life in telephinid trilobites^3^ made these arthropods an obvious prey to the orthoconic, pelagic molluscs. An unusual large number of co-occurring cephalopod species have been described^19^, and with 35 different taxa referable to 24 genera, these predators were evidently flourishing during the deposition of the Elnes Formation. Today’s representatives of cephalopods have a mouth formed like a sharp pointed horn-beak (Fig. 4a,b), comparable to the strong and effective beaks of parrots. Attacks of such beaks as these surely would produce the kind of dent-like traces that are found here. Inside the beaks, cephalopods possess a strong radula, as is part of the mouth of all typical molluscs. This radula is the cover of a muscular tongue, built of a horn-like material, and well equipped with hooks and spines (Fig. 4c–f). These structures are able to cause severe mangling when the radula is rasped over organic tissue, and may have caused the ruptures of the visual surface as the beak’s attack may have formed the cavities and ruptures in the compound eye. The smooth surface, which resulted from the (probably immediately) following healing process, resembles a repair arising from a gel-like complex produced by coagulated proteins in a clotting process, as we know it from wound plugs of modern-day crustaceans (Fig. 4j–l), and likewise vertebrates.

There are many described examples showing how effective these healing mechanisms actually were. Repaired injuries to the exoskeleton in trilobites from the early Cambrian and onwards, have been discussed amongst others by Burling^20^, Owen^21^,^22^, Owen & Tilsley^23^, Conway Morris & Jenkins^24^, Ludvigsen^25^, Babcock^26^, Rabano & Arbizu^27^, Senosiáin^28^ and Fatka *et al*.^29^. Abnormalities of the exoskeleton have been discussed and summarized by Whittington^30^, who gives further references. He commented, as did Owen^22^, that it is not always possible to distinguish whether an abnormality is the result of predation, genetic or developmental malfunction or parasitism. Occular regeneration, however, in trilobites is highly unusual, and has been reported so far just once by Isberg in 1917. The injuries to the eye of *Telephina* could, as we have seen, not easily be attributed to any other cause than an attack by a predator. Furthermore, importantly we can document here not just the final outcome, but the results of a sequence of events of a healing process following a serious injury in this ancient Ordovician arthropod, telling also a dramatic struggle for life with the result that the trilobite was able to live and fight another day.

Trilobites, such as *Hollardops mesocristata* (Le Maître)^31^, sometimes lack one of the genal spines or part of it, which has become partly regenerated as Fig. 4 shows. Thus, even a reduced regeneration of exoskeletal structures is possible. Immune and healing systems like these are very fundamental and necessary for survival, not only for an individual, but also as species during the long process of evolution. By the results shown here and in other papers, it may be assumed that the principles of immune repair systems of injuries/lesions are more than half a billion years old.

## Methods and Material

Trilobites: *Telephina intermedia* (Thorslund)^1^, [PMO 231.360, Palaeontological Museum, University of Oslo] and *Telephina bicuspis* (Angelin)^32^, [ PMO 231.361] come from dark, limestone concretions (Middle Ordovician Elnes Formation of the Darriwillan age (~465 Ma), in the northern districts of the Oslo Region, and were collected and prepared by M.H. Both specimens are housed at the Natural History Museum, Oslo, Norway. *Hollardops mericristata* (Le Maître)^31^, originates from Jbel Zguilma near Foum Zguid, Morocco, Middle Devonian [PMO 231.467]. Terms applied to trilobite morphology follow Whittington & Kelly^33^. The photographs of Figs 1a,d,f–h, 2 and 3a,b,d,e were taken with a Keyence digital microscope (VHX-900F, VH-Z20R/RZ x20-x200.) at the Steinmann Institute University of Bonn, Figs 1c,d and 3c were made with a Nikon SMZ 18 housed at the Department of Genetics at the University of Cologne, and the photographs of Fig. 4 with a Keyence microscope (VHX-900F, VH-Z20W/VHX-J20) at the Institute of Biology Education, University of Cologne.

## Additional Information

**How to cite this article**: Schoenemann, B. *et al*. Traces of an ancient immune system–how an injured arthropod survived 465 million years ago. *Sci. Rep.* **7**, 40330; doi: 10.1038/srep40330 (2017).

**Publisher's note:** Springer Nature remains neutral with regard to jurisdictional claims in published maps and institutional affiliations.
